# Changes in Medical Schools

**Published:** 1859-05

**Authors:** 


CHANGES IN MEDICAL SCHOOLS.—Pennsylvania Medical College: We learn that Dr. Stillé, who for a number of years so ably filled the chair of Medicine in this institution, retired from it a short time ago. As yet no successor has been appointed.

Dr. E. Geddings has again become united with the Medical College of South Carolina, having been appointed to the chair of Medicine, rendered vacant by the death of Dr. Gaillard.

In the Albany Medical College, Professors Hun and Dean have resigned their chairs.

Professor Townsend unites Physiology and Materia Medica, and Professor Quackenbush lectures on Obstetrics and Medical Jurisprudence.

Professor M. A. Johnston has resigned the Chair of Physiology and Pathology in the Rush Medical College of Chicago. We have not heard of the appointment of his successor.

Professor Sandford B. Hunt has resigned the Chair of General and Descriptive Anatomy and Physiology in the University of Buffalo. The chair has been divided, and Dr. Sandford Eastman has been appointed Professor of Anatomy, and Dr. Austin Flint, Jr., Professor of Physiology and Microscopic Anatomy. We congratulate the faculty upon these appointments, especially of the latter gentleman, who, although a recent graduate, is well known by his contributions to medical periodical literature, and as the editor of the Buffalo Medical Journal. As a hard worker in his profession, with high ability and attainments, we can safely predict for him a career of future honor and usefulness.

